# Current perspectives for metabolomics and lipidomics in dyslipidemia of acne vulgaris: a mini review

**DOI:** 10.3389/fmed.2024.1538373

**Published:** 2025-01-15

**Authors:** Liang Wu, Sheng-Cai Zhu, Yang He, Yun-Xia Zhu, Xiao-Liang Ou-Yang, Deng Zhang, Chun-Ming Li

**Affiliations:** ^1^Department of Dermatology, The Second Affiliated Hospital, Jiangxi Medical College, Nanchang University, Nanchang, China; ^2^Department of Plastic Surgery, The Second Affiliated Hospital, Jiangxi Medical College, Nanchang University, Nanchang, China; ^3^Department of Dermatology, The Fifth People's Hospital Affiliated to Chengdu University of Traditional Chinese Medicine, Chengdu, China

**Keywords:** acne vulgaris, dyslipidemia, metabolomics, lipidomics, sphingomyelins, phosphatidylcholines

## Abstract

Acne vulgaris (AV) is a common inflammatory disorder involving the pilosebaceous unit. Many studies have reported that people with AV have higher levels of total cholesterol (TC), triglycerides (TG), and low-density lipoprotein cholesterol (LDL-c) compared to healthy controls. Hence, they concluded that an unhealthy lipid profile is an independent risk factor for AV. Recent research in metabolomics and lipidomics has been propelled by rapid advancements in technologies including computational methods and mass spectrometry. Using metabolomics and lipidomics approach, a broad range of structurally diverse lipid species were detected and important lipid biomarkers were identified that are vital to the pathogenesis of AV. In this review, we will describe the recent progress in dyslipidemia of AV using metabolomics and lipidomics advances. We will begin with a literature overview of dyslipidemia of AV, followed by a short introduction of metabolomics and lipidomics. Finally, we will focus on applying metabolomics and lipidomics in dyslipidemia of AV.

## 1 Background

Acne vulgaris (AV), a chronic inflammatory disorder of the pilosebaceous unit, impacts 80–90% of teenagers and young adults globally between the ages of 12 and 25 years, although it can affect individuals in any age group (1, 2). Its primary clinical manifestations include inflammatory lesions (papules, pustules, nodules, and cysts) as well as non-inflammatory (comedones). Severe cases of acne can be painful and may result in scarring and disfiguration, negatively impacting one’s appearance and self-esteem, and leading to psychological distress. The progression of acne is determined by four key pathogenic factors: increased sebum production (seborrhea), follicular hyperkeratinization, colonization by *Cutibacterium acnes*, and inflammatory processes (3, 4). Additionally, various factors including genetic predisposition, diet, smoking habits, mental health, as well as seasonal and geographic influences may contribute to the development of acne (5–9). Recently, there has been growing recognition of the importance of both quantitative and qualitative changes in lipids as significant contributors to acne pathogenesis.

Lipids represent a category of biomolecules that play important biological roles, serving as structural elements of cellular membranes and microdomains, participating in energy metabolism, and acting as signaling intermediates in signaling pathways (10). These molecules exhibit dynamic characteristics and are significantly affected by both exogenous or endogenous factors, such as pharmacological agents, genetic predispositions, lifestyle choices, dietary habits, age, and inflammation. The International Lipid Classification and Nomenclature Committee classifies lipid compounds into eight distinct groups. Each group can be further divided into various lipid classes according to their polarity. Moreover, variations in the saturation or length of carbon chains facilitate further classification into specific lipid species (11). An imbalance of lipid metabolism is associated with numerous health conditions, including hypertension, diabetes, obesity, metabolic syndrome, and cancer (12).

Traditionally, dyslipidemia is often defined by elevated levels of total cholesterol (TC), low-density lipoprotein cholesterol (LDL-c), and triglycerides (TG), alongside reduced levels of high-density lipoprotein cholesterol (HDL-c) in the bloodstream. Nonetheless, findings across various studies remain inconsistent. For instance, one investigation involving 530 individuals with AV and 550 controls without AV examined lipid profiles, revealing that those with AV had lower levels of HDL-c compared to their counterparts without AV (13). Conversely, another study reported that HDL-c levels were notably higher in patients with AV (14). Consequently, this study aims to further analyze and understand the connection between acne and dyslipidemia.

## 2 Literature review of dyslipidemia of acne vulgaris

### 2.1 Acne vulgaris

In most studies, dyslipidemia was found among patients with AV (13–26). For instance, in a case–control study conducted on a large cohort from the Pakistan population, Younis and colleagues reported significant increases in TC and TG levels among patients with acne. Conversely, the study also noted a marked reduction in HDL-c levels in these individuals (13). Similarly, additional research from populations in Saudi Arabia and Egypt demonstrated elevated TG, TC, and LDL-c levels in patients suffering from acne (15, 19, 23). Notably, there are few studies focusing on blood profiles of severe AV. A study conducted by Arora et al. found that the levels of TC, LDL-c, and HDL-c were substantially higher in female patients with severe AV compared to their counterparts (26). Furthermore, a retrospective research by Jiang et al. revealed that both male and female patients with severe AV also exhibited significantly elevated levels of TC and LDL-c when compared to healthy control subjects (16). However, it is important to note that not all studies have established a link between acne and dyslipidemia. For instance, Akl et al. found no significant differences in the lipid profiles of TG, TC, LDL-c, and HDL-c levels between patients with AV and healthy control subjects (27).

### 2.2 Acne of different ages

#### 2.2.1 Pediatric acne

The categorization of pediatric acne based on age was performed following the guidelines set by Eichenfield et al. They classified pediatric acne into several groups: neonatal acne (from birth to 6 weeks), infantile acne (from 6 weeks to 1 year), mid-childhood acne (ages 1 to 7), preadolescent acne (from 7 to 12 years or before girls reach menarche), and adolescent acne (ages 12 to 19 or after menarche in girls) (28).

A cross-sectional observational study was carried out by Pareek and colleagues over an 18-month period, involving 50 children aged 1 to 12 years diagnosed with acne. The findings indicated that 19 of the 50 participants (36%) exhibited abnormalities in their lipid profiles. Additionally, the study established a significant positive relationship between HDL-c levels and the severity of acne, whereas no significant association was observed between LDL-c, TG, or TC levels and acne severity (29). Abulnaja categorized adolescent females into four distinct groups: those who are obese with acne, non-obese with acne, obese without acne, and non-obese without acne. He observed that obese adolescents suffering from acne displayed significantly elevated levels of serum TG and LDL-c compared to their obese counterparts without acne, as well as to the non-obese group; however, their HDL-c levels were notably lower (30).

#### 2.2.2 Postadolescent acne

Postadolescent acne, namely adult acne, has been traditionally defined as the occurrence of acne after the age of 25 years. To investigate the lipid levels in patients with postadolescent acne, Ekiz et al. recruited 184 acne patients and 82 healthy controls in Turkey and found that only HDL-c levels were significantly decreased with postadolescent acne (31). Another study in a Turkey population demonstrated that both TC and LDL-c levels were notably elevated in individuals suffering from postadolescent acne (32). Furthermore, a case–control study conducted by Abdel Rahman et al. within an Egyptian population revealed that serum levels of TG were significantly increased in the postadolescent acne patients group compared to the control. However, serum TC, HDL-c, and LDL-c showed no significant difference between the studied groups (33).

The previous studies on AV and dyslipidemia have been summarized in Table 1.

**Table 1:** Literature review of dyslipidemia of acne vulgaris.

Classification		Ref.	Patients	*n*	TC	TG	HDL-c	LDL-c
Acne vulgaris		(15)	Saudi Arabia	144	↑	↑	–	↑
		(13)	Pakistan	1,080	↑	↑	↓	NA
		(16)	China	311	↑	↑^#^	–	↑
		(14)	Saudi Arabia	200	↑	↑	↑	↑
		(17)	Egypt	120	↑	–	↓	↑
		(18)	Poland	88	NA	↑	–	NA
		(19)	Egypt	80	↑	↑	–	↑
		(20)	Jordan	271	–	–	↓	–
		(21)	Iran	80	–	–	↓	–
		(22)	India	120	↑	–	↓	↑
		(23)	Saudi Arabia	106	↑	↑	–	↑
		(27)	Egypt	120	–	–	–	–
		(24)	India	70	–	↑	–	–
		(25)	Iran	90	↑	–	–	–
		(26)	India	180	↑	–	↑	↑
Pediatric acne	Mid-childhood acne and preadolescent	(29)	India	50	–	–	–	–
	Adolescent acne	(30)	Saudi Arabia	60	NA	↑	↓	↑
Postadolescent acne		(33)	Egypt	100	–	↑	–	–
		(31)	Turkey	266	–	–	↓	–
		(32)	Turkey	70	↑	–	–	↑

*n, a total number of acne patients and controls; TC, total cholesterol; TG, triglycerides; HDL-c, high-density lipoprotein cholesterol; LDL-c, low-density lipoprotein cholesterol. “↑” indicates a significant increase. “↓” indicates a significant decrease. “–” indicates no significant difference. “NA” indicates that no detection is performed. “#” indicates that TG are significantly elevated in male patients suffering from severe acne.*

## 3 Metabolomics and its application in dyslipidemia of AV

### 3.1 Metabolomics

Metabolomics is an emerging field that follows the development of genomics, transcriptomics, and proteomics analysis (34, 35). It encompasses the study of all metabolic responses of living organisms to genetic modifications of external substances, stimuli, or environmental alterations. Researches in metabolomics concentrate on small molecular metabolites including lipids, small molecular peptides, amino acids, and organic acids (molecular weight < 1,000 Da) (36, 37). Through this comprehensive study, researchers are able to gain insights into these responses along with their dynamic alterations.

In recent years, metabolomics has emerged as a powerful tool in various kinds of medical research, encompassing areas such as early diagnosis, personalized treatment strategies, evaluations of drug efficacy and toxicity, as well as drug target screening (38). The advantages of metabolomic technology include the following: firstly, it does not necessitate whole-genome sequencing or the development of a gene database. Secondly, small yet significant changes in gene and protein expression are reflected in metabolites, facilitating easier detection. Thirdly, the types and quantities of metabolites are considerably less than those of genes and proteins. Lastly, body fluids such as sweat, saliva, tears, and urine can be used by metabolomics as substrates, enhancing the convenience of sampling. Nonetheless, metabolomics also presents certain limitations, including low qualitative and quantitative accuracy, inadequate detection sensitivity, restricted metabolite coverage, and so on (39).

Research technologies in metabonomics primarily encompass liquid chromatography-mass spectrometry (LC–MS), gas chromatography–mass spectrometry (GC–MS), and nuclear magnetic resonance (NMR), among others. Notably, NMR represents the earliest application in this field, while MS is regarded as the most commonly utilized and effective technology (40, 41). Metabolomic analysis is categorized into two approaches: non-targeted and targeted methods. The non-targeted metabolomic approach aims to identify all metabolites present in the sample. Conversely, the targeted metabolomic method involves the prior determination of specific biomarkers, which are then validated throughout the analysis (42).

### 3.2 Application of metabolomics in dyslipidemia of AV

In individuals with AV, alterations occur in serum metabolite composition. A Mendelian randomization study conducted by Wang et al. revealed the causal relationship between serum metabolites and AV (43). Besides, several studies have reported the application of metabolomics in skin tissues of acne animal models (34, 44). However, there are few metabolomic studies focusing on blood samples from acne patients. Yu et al. employed LC–MS/MS to analyze the plasma samples from individuals suffering from moderate to severe acne. Their finding identified 63 significant differential metabolites. Notably, four sphingolipid metabolites including sphinganine, sphingosine, O-Phosphoethanolamine, and sphingomyelin (SM) (d18:1/18:0) were found to be upregulated (45). It has been reported that insulin resistance is prevalent among the majority of patients with acne. Li et al. utilized LC–MS/MS to analyze serum metabolites from patients with acne, differentiating those with insulin resistance from those without. They identified several lipid metabolites, including 1,2-Dioleoyl-sn-glycero-3-phosphatidylcholine and SM (d18:1/18:0), as being downregulated. Additionally, this research team noted that the sphingolipid signaling pathway was recognized as one of the eight pathways significantly enriched according to KEGG analysis (46).

## 4 Lipidomics and its application in dyslipidemia of AV

### 4.1 Lipidomics

Lipidomics, which was initially introduced in 2003, is a subfield of metabolomics (47). It enables a thorough and systematic analysis of lipids in cells, tissues, or organisms and the molecules that interact with them. This field aims to elucidate the structure and function of lipids, thereby uncovering the connections between lipid metabolism and the physiological as well as pathological processes occurring in cells, organs, and the entire body. Currently, lipidomics research primarily focuses on: (1) Employing high-throughput techniques and molecular modeling utilizing lipidomics data; (2) Determining the structure and classification of novel lipids; and (3) Conducting network analyses to clarify metabolic processes in both healthy and diseased individuals, including biomarker assessments of disease conditions (48).

The lipidomics research methodologies encompass various techniques, such as NMR, chromatography, ultra-performance liquid chromatography-quadrupole time-of-flight mass spectrometry (UPLC-QTOF-MS), and electrospray ionization mass spectrometry (ESI-MS), etc. In lipidomics analysis, there are two main approaches: untargeted and targeted. Untargeted lipidomics entails an unbiased, comprehensive examination of all identifiable lipids within a sample, whereas targeted lipidomics is centered on accurately measuring specific lipid molecules (49, 50).

### 4.2 Application of lipidomics in dyslipidemia of AV

Lipidomics has been studied in numerous dermatologic diseases, including hidradenitis suppurativa, atopic dermatitis, psoriasis, and eczema (51–54). In the realm of acne research, several lipidomics studies have demonstrated an association between alterations in skin lipid metabolites and acne (55–59). Nonetheless, limited lipidomic studies focused on analyzing blood samples from acne patients. Zhang et al. conducted an untargeted lipidomics analysis of plasma from patients with moderate-to-severe AV. They found significant differences in plasma lipid profiles between the patients with AV and the control group. Moreover, 26 significantly different lipid metabolites were identified. The primary constituents of these lipid metabolites were SMs (*n* = 7)including SM (d34:1), SM (d36:2), SM (d36:1), SM (d38:1), SM (d42:1), SM (d42:3), and SM (d42:2), and phosphatidylcholines (PCs) (*n* = 4) including PC (8:1e_10:1), PC (16:0_20:3), (PC) (34:1), PC (36:1) (60). Additionally, targeted lipidomics of serum sphingolipids in acne patients has been conducted by Kaya et al. They utilized an optimized multiple reaction monitoring (MRM) technique in combination with ultra-fast liquid chromatography (UFLC) and tandem mass spectrometry to quantify serum concentrations of C16-C24 SMs and C16-C24 ceramides (Cers). They observed patients with AV had increased circulating levels of C16 SM and lower circulating levels of C24 Cer compared to healthy controls, which may provide prognostic value for the disease (61).

### 4.3 Major different lipid metabolites identified by lipidomics in acne pathogenesis

Results from the lipidomics study revealed that the significantly different lipid metabolites primarily consisted of SMs and PCs (60). Therefore, we will discuss the significance of these lipid metabolites in the development of AV.

#### 4.3.1 The role of sphingolipid in AV pathogenesis

SM is the most abundant circulating lipid. Within cells, it plays a crucial role in the formation of lipid rafts and structured membrane microdomains (62, 63). An *in vitro* study demonstrated that SM can influence Toll-like receptor 4 signaling in macrophages, mediating the inflammatory response (64). Additionally, it can stimulate the proliferation of keratinocytes and inhibit their differentiation (40). Consequently, elevated levels of SM in plasma may contribute to the pathogenesis of acne by regulating abnormal keratin differentiation and inflammatory processes.

Under normal physiological conditions, SM can be hydrolyzed by sphingomyelinase to produce Cer, which can subsequently be regenerated into SM through the action of sphingomyelin synthase, a process referred to as the SM cycle (41). The increase in SM in plasma from individuals with acne may lead to an abnormal accumulation of Cer. *In vitro* studies indicate that Cer plays a crucial role in regulating the balance between keratinocyte proliferation and differentiation by inhibiting cell proliferation while promoting apoptosis (65). Furthermore, disturbances in Cer metabolism can impair barrier function, which in turn leads to the sustained production of cytokines and chemokines, including interleukin-1*α* (IL-1α), tumor necrosis factor-α (TNF-α), and *β*-defensins, thereby exacerbating the inflammatory response (66).

#### 4.3.2 The role of phosphatidylcholine in AV pathogenesis

As the primary component of eukaryotic cell biofilms, the biosynthesis and degradation of PC are vital for regulating the cell cycle process and its synthetic deficiency serving as a marker of apoptosis (67). Additionally, PC has been shown to improve experimental arthritis and modulate lipopolysaccharide-induced inflammatory responses by regulating leukocyte activation and maintaining the balance of the gut-brain axis (68, 69). In *in vitro* experiments, PC can inhibit inflammatory responses induced by TNF-*α* and IL-6 in macrophages and monocytes. This effect may be related to PC’s inhibition of the activation of mitogen-activated protein kinases (MAPKs) / ERK and p38 signaling pathways, thereby preventing the transport of the nuclear transcription factor kappa B (NF-kB) (70, 71). Previous studies have demonstrated that various types of PC can regulate the differentiation of keratinocytes. Hence, alterations in PC levels may lead to abnormal keratin differentiation and inflammation in the pathogenesis of acne.

Besides, the removal of PC fatty acid chains at the sn-2 position by cytosolic phospholipase A2 leads to the formation of lysophosphatidylcholine (LPC) (72). *In vitro* studies have shown that LPC exerts various stimulatory effects on immune cells, including monocytes, macrophages, T lymphocytes, and neutrophils (73). Furthermore, research indicates that LPC can activate the NF-kB, p38MAPK, and JUN signaling pathways, which in turn induces the production of pro-inflammatory factors such as IL-1β and IL-8, playing a crucial role in the regulation of acne inflammation (74, 75).

## 5 Conclusion and future perspective

As noted in most studies, dyslipidemia has been observed in patients with AV. Utilizing metabolomics and lipidomics approaches, potential lipid markers such as SMs and PCs have been identified. However, transcriptomics and proteomics have not yet been employed to verify the expression levels of the related mRNA and proteins. Therefore, the integration of multiple omics disciplines (including metabolomics, lipidomics, genomics, transcriptomics, and proteomics) is essential (76). This not only enhances the requirements for researchers but also raises expectations for further investigations into the pathogenesis and treatment of acne.
